# Life Course Dietary Patterns and Bone Health in Later Life in a British Birth Cohort Study

**DOI:** 10.1002/jbmr.2798

**Published:** 2016-03-08

**Authors:** Kate A Ward, Ann Prentice, Diana L Kuh, Judith E Adams, Gina L Ambrosini

**Affiliations:** ^1^ MRC Human Nutrition ResearchCambridgeUK; ^2^ MRC Unit for Lifelong Healthy Ageing at University College LondonLondonUK; ^3^ Manchester Academic Health Sciences & University of ManchesterCentral Manchester University Hospitals NHS Foundation TrustManchesterUK; ^4^ School of Population HealthThe University of Western AustraliaPerthAustralia

**Keywords:** DIETARY PATTERNS, BONE, LONGITUDINAL, DUAL ENERGY X‐RAY ABSORPTIOMETRY, REDUCED RANK REGRESSION

## Abstract

Evidence for the contribution of individual foods and nutrients to bone health is weak. Few studies have considered hypothesis‐based dietary patterns and bone health. We investigated whether a protein‐calcium‐potassium–rich (PrCaK‐rich) dietary pattern over the adult life course, was positively associated with bone outcomes at 60 to 64 years of age. Diet diaries were collected at ages 36, 46, 53, and 60 to 64 years in 1263 participants (661 women) from the MRC National Survey of Health and Development. DXA and pQCT measurements were obtained at age 60 to 64 years, including size‐adjusted bone mineral content (SA‐BMC) and volumetric bone mineral density (vBMD). A food‐based dietary pattern best explaining dietary calcium, potassium, and protein intakes (g/1000 kcal) was identified using reduced rank regression. Dietary pattern *Z*‐scores were calculated for each individual, at each time point. Individual trajectories in dietary pattern *Z*‐scores were modeled to summarize changes in *Z*‐scores over the study period. Regression models examined associations between these trajectories and bone outcomes at age 60 to 64 years, adjusting for baseline dietary pattern *Z*‐score and other confounders. A consistent PrCaK‐rich dietary pattern was identified within the population, over time. Mean ± SD dietary pattern *Z*‐scores at age 36 years and age 60 to 64 years were –0.32 ± 0.97 and 2.2 ± 1.5 (women) and –0.35 ± 0.98 and 1.7 ± 1.6 (men), respectively. Mean trajectory in dietary pattern *Z*‐scores ± SD was 0.07 ± 0.02 units/year. Among women, a 0.02‐SD unit/year higher trajectory in dietary pattern *Z*‐score over time was associated with higher SA‐BMC (spine 1.40% [95% CI, 0.30 to 2.51]; hip 1.35% [95% CI, 0.48 to 2.23]), and vBMD (radius 1.81% [95% CI, 0.13 to 3.50]) at age 60 to 64 years. No statistically significant associations were found in men. During adulthood, an increasing score for a dietary pattern rich in protein, calcium, and potassium was associated with greater SA‐BMC at fracture‐prone sites in women. This study emphasizes the importance of these nutrients, within the context of the whole diet, to bone health. © 2016 The Authors. *Journal of Bone and Mineral Research* published by Wiley Periodicals, Inc. on behalf of American Society for Bone and Mineral Research (ASBMR).

## Introduction

The contribution of environment to bone health has long been described. As a key component of environment, the role of diet has been tested in cross‐sectional and longitudinal studies at different stages of the life course and the potential impact of multiple dietary factors on bone health and fracture risk has been reported.^1^ Despite this, there is a lack of consistency between studies; there are multiple reasons for this: (1) there are few, if any, studies of the cumulative effects of diet through the life course; (2) a single dietary nutrient is a small part of total intake and dietary composition; (3) diet also reflects socioeconomic status and lifestyle of the individual; and (4) randomized controlled trials of diet only last 1 to 2 years, which may not be long enough to show an effect.^2^, ^3^, ^4^

To build on what is already known, dietary data collected at multiple time points from longitudinal cohort studies provide an ideal opportunity to study how diet might contribute to skeletal health through the life course. Choosing the appropriate method of analysis is important to fully exploit such data. Approaches that consider the diet as a whole, through dietary pattern analysis, may be beneficial, not least because they can be translated into food‐based public health messages about overall diet, which may be easier for the public to interpret and implement.^2^ In comparison to analyzing data in terms of individual foods or nutrients, dietary patterns have the advantage of taking account of total intake and dietary composition and the potential additive effects between foods and nutrients consumed together rather than focusing only on a single nutrient or food group.

All but one^5^ of the studies published to date on dietary patterns and bone health have been cross‐sectional and relied on exploratory, data‐driven approaches (eg, principal components analysis) to identify dietary patterns.^5^, ^6^, ^7^, ^8^, ^9^, ^10^, ^11^, ^12^ A “nutrient dense” dietary pattern rich in nutrients but not energy, characterized by high intakes of fruit, vegetables, and whole grains has been associated with higher bone mineral density or content (BMD/BMC) and reduced fracture risk in several studies.^5^, ^6^, ^7^, ^9^, ^10^, ^13^, ^14^, ^15^, ^16^, ^17^, ^18^, ^19^ (Nutrient‐dense dietary patterns have been defined in publications from the U.S. Department of Agriculture^20^ and the National Institutes of Health.^21^) Conversely, dietary patterns characterized by intakes of combinations of foods, including confectionery, soft drinks, processed meats, and biscuits (cookies), have been negatively associated with BMD/BMC.^6^, ^7^, ^9^

An alternative to purely exploratory dietary pattern methods is reduced rank regression (RRR), which incorporates a priori information to identify hypothesis‐driven dietary patterns. This has the advantage of testing hypotheses regarding specific nutrients while taking account of all foods consumed and dietary composition.

Longitudinal dietary data have been collected from the MRC National Survey for Health and Development (NSHD), a post‐war UK birth‐cohort of men and women born during 1 week in March 1946. Dietary data were collected throughout adulthood. Peripheral quantitative computed tomography (pQCT) and dual‐energy X‐ray absorptiometry (DXA) measurements conducted at age 60 to 64 years. The NSHD provides an opportunity to investigate how life course lifestyle might relate to healthy ageing and therefore the main aim of this study was to investigate how diet through adulthood might influence bone phenotype in early old age. We generated a hypothesis based on those single nutrients or food groups where there has been strong evidence for a positive role in musculoskeletal health, ie, in ameliorating bone loss or increasing BMD, and taking into account the UK diet and food supply. Those nutrients that require biomarker measurement, such as urinary sodium excretion for sodium status, were not considered. Calcium was selected as the main bone‐forming mineral, protein because of the associations reported between protein intake and bone and muscle health, and potassium as a component of the acid‐base balance and marker of fruit and vegetable intake.^1^, ^16^, ^17^, ^18^, ^22^, ^23^, ^24^, ^25^, ^26^, ^27^, ^28^, ^29^ We also took into consideration the possibility of confounding; for example, we chose potassium rather than magnesium because the strength of evidence for potassium is most consistent for bone health, and also knowing that potassium and magnesium intakes are closely correlated. Therefore adding magnesium would be unlikely to substantially improve our dietary pattern model fit. Vitamin D was not chosen because dietary intakes of vitamin D in the UK population are extremely low; with the exceptions of spreads and margarines, which at the time of the study (1940–2013) was mandatory and remains common, food fortification with vitamin D is on a voluntary basis.^30^ Therefore, RRR was used to identify dietary patterns or combinations of food intake that best characterize calcium, protein, and potassium intakes. We hypothesized that an increasing trajectory in scores for a positive dietary pattern characterized by high calcium, potassium, and protein (PrCaK‐rich) intakes during adulthood would be associated with greater BMD at 63 years of age. We also determined, at a population level, how such a dietary pattern tracked through the adult years.

## Subjects and methods

### MRC‐NSHD

The MRC‐NSHD is based on a nationally representative sample of 5362 births out of all the single, legitimate births that occurred in 1 week in March 1946 in England, Scotland, and Wales. At the 24th follow‐up, when participants were 60 to 64 years old (between 2006 and 2010),^31^ study members still alive and with a known current address in England, Scotland, or Wales were invited for an assessment at one of six clinical research facilities (CRFs) or a visit by a research nurse at home. Of those invited, 2229 (78%) were assessed: 1690 (59.2%) attended a CRF and the remaining 539 were visited at home. The participating sample remains broadly representative of native‐born British men and women of the same age.^32^

### Dietary information

Information on dietary intake was collected at follow‐up visits conducted in 1982 (at age 36 years), 1989 (age 43 years), 1999 (age 53 years) and 2006 to 2010 (age 60 to 64 years) and has been described in detail.^33^ Participants were requested to complete a 7‐day food diary at age 36 and 43 years and a 5‐day food diary at age 53 and 60 to 64 years, recording all food and beverages they consumed. Each diary was coded and linked to the contemporary British food composition tables using the MRC Human Nutrition Research (Cambridge, UK) in‐house programs (Data In Diet Out [DIDO] and Diet In Nutrients Out [DINO]) to estimate average daily nutrient intakes.^17^, ^34^, ^35^, ^36^ Whether calcium, vitamin D, multivitamin supplements, or multimineral supplements were used was available at 35 years, 53 years, and 60 to 64 years of age; however, supplement use was negligible in all but the latest follow‐up.

### Dietary patterns

In order to conduct the dietary pattern analysis, the number of recorded foods had to be reduced and these were therefore collapsed into 46 major food groups based on culinary usage and nutrient profile (see Supporting Table 1). RRR was applied to identify dietary patterns best associated with high protein, calcium, and potassium densities (amount consumed relative to energy intake). RRR is useful for identifying combinations of food intakes or dietary patterns that explain the maximum variation in a set of response variables. The response variables are selected on the basis that they are hypothesized to be on the causal pathway between food consumption and the health outcome of interest.^37^ The RRR model therefore included all 46 food groups (g/day) as predictors and average nutrient densities of protein (% of total energy), calcium (g/1000 kcal), and potassium (g/1000 kcal) as response variables. Because we were interested in dietary patterns that explain the most variation in nutrient intakes from food and available supplement use data was categorical (yes/no), supplement use was not included in the RRR models. Separate RRR models were first run for each follow‐up, using the PLS procedure in SAS (SAS Institute, Cary, NC, USA). Owing to only minor differences in their dietary patterns, men and women were analyzed together in the final RRR models to identify the primary dietary patterns in this population.

**Table 1:** Correlations Between Response Variables and Dietary Patterns by Survey Year

Survey year	Dietary pattern	% Explained from protein	% Explained from calcium (mg/1000 kcal)	% Explained from potassium (mg/1000 kcal)	Explained variation (%)^a^
1982	1	0.60	0.52	0.61	42.8
	2	−0.30	0.85	−0.43	15.9
	3	0.75	−0.07	−0.66	7.0
1989	1	0.54	0.58	0.62	44.6
	2	−0.32	0.81	−0.48	13.6
	3	0.78	−0.06	−0.62	8.7
1999	1	0.51	0.58	0.63	46.3
	2	0.51	−0.80	0.33	12.9
	3	0.70	0.15	−0.70	8.9
2006–2010	1	0.51	0.57	0.64	42.6
	2	0.49	−0.81	0.32	11.9
	3	0.70	0.16	−0.70	8.8

^a^ Total variation in response variables explained by the dietary pattern.

Because three response variables were included in the RRR models, three dietary patterns were identified for each follow‐up. The first dietary pattern was consistently positively correlated with protein, calcium, and potassium intakes and explained the most variation in all three response variables (43% to 46%) in all survey years (Table 1), whereas the second and third dietary patterns each explained considerably less variation in all response variables (7% to 16%) and were less consistent over survey years. For these reasons, and because the first dietary pattern corresponded with our hypothesized dietary pattern, it was the only dietary pattern taken forward and investigated in relation to bone outcomes. At each time point, the study participant received a *Z*‐score for the first dietary pattern, indicating how closely their reported dietary intake reflected the dietary pattern relative to others in the study population.

**Figure 1:**
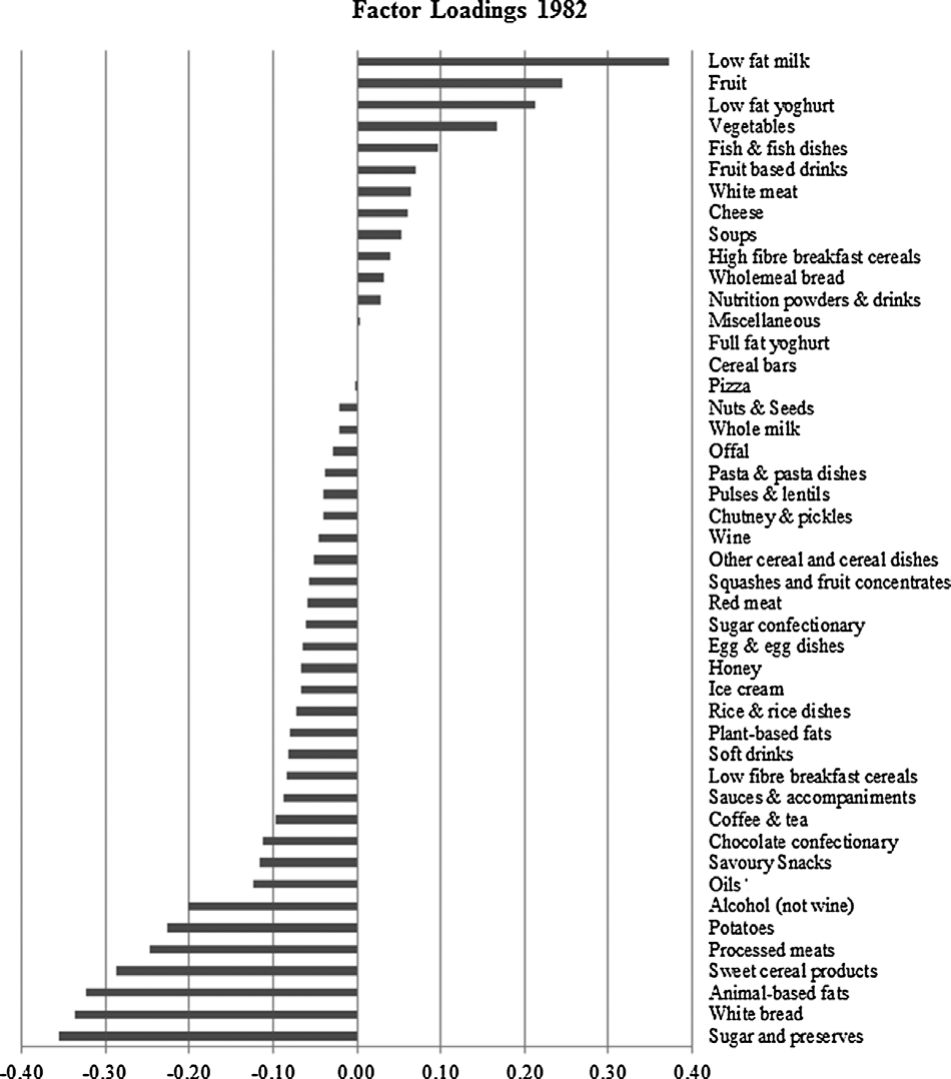
Factor loadings for the protein‐calcium‐potassium–rich dietary pattern in 1982.

The foods and their factor loadings characterizing the PrCaK‐rich dietary pattern at age 36 years are shown in Fig. 1 (see Supporting Fig. 1 for other follow‐ups). A positive factor loading indicates that greater consumption of that food by an individual increases the individual's *Z*‐score for the dietary pattern, and a negative factor loading indicates that greater consumption of that food decreases the *Z*‐score.

Although the foods represented in the PrCaK‐rich pattern were similar across survey years, there were some minor differences (see Supporting Fig. 1). Therefore, to assess longitudinal changes in *Z*‐scores for exactly the same dietary pattern, confirmatory RRR was used to apply the dietary pattern identified in 1982, prospectively, across all survey time points. This involved applying the scoring weights for dietary pattern in 1982 to food intakes recorded in 1989, 1999, and 2006. As a result, each study participant received a *Z*‐score for the first dietary pattern in 1982, 1989, 1999, and 2006 (where food diaries were available).

### Bone densitometry

Details of the scanning protocols have previously been described.^38^ In brief, 1690 participants attended one of the six CRFs and 1658 had DXA (QDR 4500 Discovery; Hologic Inc, Bedford, MA, USA) scans of the whole body, hip, or spine. pQCT (XCT 2000; Stratec Medizintechnik, Pfrozheim, Germany) equipment was only available in five CRFs so fewer individuals (*n* = 1350) had a pQCT scan of the nondominant radius (distal 4% and diaphyseal 50% sites). All sites followed a standardized scanning protocol and were trained by the lead data collection center in Manchester.

DXA scans were analyzed using software version APEX 4.1. Whole body, lumbar spine (L_1_–L_4_), and total hip data were used in this study. We present DXA data as size‐adjusted BMC rather than areal BMD (aBMD) because preliminary analysis showed that the power coefficients for the relationship between BMC and bone area (BA) at these sites were greater than 1, indicating that using aBMD would not correct BMC entirely for differences in BA, leading to underestimation or overestimation of BMD in participants of different sizes.^39^

pQCT scans were analyzed using manufacturer software (version 6.0): contour mode 2, peel mode 1 were used for the distal radius; for the diaphysis separation mode 1 threshold 710 mg/cm^3^ was used for all outcomes except the stress strain index (SSI), where a threshold of 480 mg/cm^3^ was used, as per manufacturer recommendations. Outcomes were total and trabecular volumetric BMD (mg/cm^3^) at the distal (4%) site, and at the diaphysis (50%) site total cross‐sectional area (CSA‐dia) and cortical vBMD were used; medullary CSA was calculated by subtracting cortical area from total area. SSI was used as an in vivo estimate of bone strength (mm^3^).^40^

Machine variability between centers was monitored using the European Spine Phantom and the pQCT scanners using the European Forearm phantom; where necessary, cross‐calibration was performed.^41^, ^42^, ^43^, ^44^ Standard manufacturer procedures were followed for daily quality assurance (QA)/quality control (QC) and all phantom and scan analysis were centralized to one center (Manchester Academic Health Sciences & University of Manchester, Central Manchester University Hospitals NHS Foundation Trust, Manchester, UK; JEA) for grading, analysis, and collation of a harmonized database. Repeat precision was determined in one center and was <1% for DXA measurements; for pQCT it ranged between 1% and 3%.

### Anthropometry and other covariates

Height and weight were measured by trained research nurses using the same standardized protocols at each age that the dietary assessments were made. For the last time point, weight and height were measured at the time of bone mineral measurement. Information on prescribed oral glucocorticoids, aromatase inhibitors, and all medications taken for osteoporosis was obtained at age 60 to 64 years.^31^ Time of natural menopause or hysterectomy/bilateral oophorectomy was determined using data obtained annually from ages 47 to 54, and at ages 57 and 60 to 64 years (*n* = 709).^45^ Smoking status, physical activity level (by nurse‐led questionnaire^46^), social class (based on occupation) from age 53 years were used as potential confounders,^47^ as was geographical region of residence at age 36 years.^48^

### Statistical analysis

#### Descriptive analyses

Mean ± SD for musculoskeletal phenotypes and key dietary intakes and confounding variables were calculated to describe their distributions. To provide a full description of the PrCaK‐rich dietary pattern, mean ± SD intakes of a range of nutrients were calculated according to quintiles of the dietary pattern scores. Trends in nutrient densities (g/1000 kcal) were estimated by modeling the dietary pattern *Z*‐score quintile as a categorical independent variable against nutrient density.

#### Tracking of the dietary pattern

The overall stability—or tracking of *Z*‐scores for the PrCaK‐rich dietary pattern over the life course and at the population level—was assessed by estimating a tracking coefficient. Using a generalized estimating equation (GEE) model in STATA (StataCorp, College Station, TX, USA), the baseline dietary pattern *Z*‐score was regressed as an independent variable against all subsequent dietary pattern *Z*‐scores measured during follow‐up, as outcomes, adjusting for the time between measurements, social class, and geographic region. Men and women were analyzed separately and those with more than one completed food diary (*n* = 1418) were included in the analysis. The standardized regression coefficient for the baseline dietary pattern *Z*‐score was interpreted as the tracking coefficient, which reflects the longitudinal association between the first dietary pattern *Z*‐score and subsequent *Z*‐scores. The tracking coefficient typically has a value between 0 and 1, the closer the coefficient to 1, the stronger the tracking; a value of 1 would indicate perfect tracking, ie, exactly the same dietary pattern *Z*‐score over time. Tracking coefficients may be influenced by measurement error or the length of time between measurements; however, a coefficient ≥0.4 was chosen a priori to signify moderate tracking.

#### Individual trajectories in dietary pattern scores between ages 36 and 60 to 64 years

Of the 1569 study participants with DXA measurements at ages 60 to 64 years, 151 completed one food diary, 246 completed two, 400 completed three, and 772 completed four food diaries between 1982 and 2006–2010. To summarize changes in *Z*‐scores for the PrCaK‐rich dietary pattern over the life course while utilizing all available diet diaries, trajectories in dietary pattern *Z*‐scores were modeled for each study participant who completed more than one diet diary (*n* = 1418). The dietary pattern trajectory is similar to a regression line or slope, which summarizes the longitudinal development or changes in an individual's dietary pattern *Z*‐scores over time. Dietary pattern trajectories were estimated by modeling all available dietary pattern *Z*‐scores (1982 to 2010) against time using a linear mixed effects model (“xtmixed” in STATA) that included random effects for the intercept (person) and time (age). This allowed both the baseline dietary pattern *Z*‐score (intercept) and dietary pattern trajectory (slope) to vary between individuals. Each participants' dietary pattern trajectory was estimated as the fixed effect (regression coefficient) for time, plus their individual predicted random effect for time, using best linear unbiased predictors (“BLUP”) in STATA. In order to enable direct comparisons between genders, men and women were modeled together. An interaction term for time by gender was not statistically significant (*p* = 0.05).

#### Association between life course dietary pattern trajectory and musculoskeletal phenotypes

Associations between individual trajectories in *Z*‐scores for the PrCaK‐rich dietary pattern (between age 36 years and 60 to 64 years) and bone outcomes at age 60 to 64 years were investigated using multivariate linear regression models. All bone outcomes were transformed to their natural logarithms before analysis. The basic model included dietary pattern trajectory and baseline dietary pattern *Z*‐score as independent variables. A second model adjusted for height and weight plus bone area where BMC was the outcome. A third model additionally adjusted for social class, geographical region, physical activity, cigarette smoking, supplement use, and time since menopause (females) (see Table 5 for numbers of participants in each analysis). We decided a priori to analyze associations between dietary pattern trajectories and bone outcomes in men and women separately because of the known sexual dimorphism in the timing of age‐related versus menopausal bone loss and in fracture risk.

## Results

Distributions of cohort characteristics, bone outcomes, and key dietary variables used in this analysis are presented in Table 2 and Table 3. A total of 1263 individuals (661 women) had dietary information at more than one time point and bone measurements at age 60 to 64 years.

**Table 2:** Cohort characteristics

	Women		Men	
	*n*	Mean ± SD	*n*	Mean ± SD
Characteristics at age 60–64 years				
Age (years)	758	63.1 ± 1.1	678	63 ± 1.1
Height (m)	758	1.62 ± 0.06	678	1.75 ± 0.07
Weight (kg)	758	71.8 ± 14	678	84.8 ± 13
BMI (kg/m^2^)	758	27.3 ± 5.1	678	27.5 ± 3.9
Time since menopause (years)	709	13.7 ± 5.3	678	–
	*n*	%	*n*	%
Social class at age 53 years^a^				
I	18	2.6	98	15.5
II	309	44.2	299	47.2
III non‐manual	242	34.6	71	11.2
III manual	45	6.4	120	18.9
IV	65	9.3	38	6.0
V	20	2.9	8	1.3
Region of residence at age 36 years, *n* (%)				
Scotland	65	8.9	52	8.0
North, North‐West, Yorkshire	147	20.2	153	23.7
Midlands, North Midlands	142	19.5	114	17.6
South West, South	71	9.8	63	9.7
Wales	33	4.5	25	3.9
South East, London	270	37.1	240	37.1
Calcium, vitamin D, or mineral supplement user at age 60–64 years	291	38.4	174	25.7
Multivitamin or multimineral user at age 60–64 years	181	23.9	81	12.0

^a^ Social class: I = professional; II = intermediate; III non‐manual = skilled non‐manual; III manual = skilled manual; IV = semi‐skilled; V = unskilled.

**Table 3:** Bone Outcomes and Key Dietary Variables at Age 60–64 Years

	Women		Men	
	*n*	Mean ± SD	*n*	Mean ± SD
Dietary pattern (*Z*‐score)	989	2.2 ± 1.5	880	1.7 ± 1.6
Protein (% energy/day)	989	17.0 ± 3.3	880	16.4 ± 2.8
Potassium (g/1000 kcal/day)	989	1.93 ± 0.40	880	1.72 ± 0.31
Calcium (g/1000 kcal/day)	989	0.52 ± 0.1	880	0.46 ± 0.1
Total energy (kcal/day)	989	1688 ± 361	880	2085 ± 465
Trabecular density (mg/cm^3^)	600	172 ± 43	559	205 ± 43
Total density (mg/cm^3^)	600	328 ± 70	559	391 ± 68
CSA‐dia radius 50% (cm^2^)	609	1.11 ± 0.182	561	1.54 ± 0.23
Medullary area (cm^2^)	609	0.36 ± 0.16	560	0.43 ± 0.15
Polar Stress Strain Index (cm^3^)	609	2.11 ± 0.43	561	3.48 ± 0.72
BMC whole body (kg)	710	2.03 ± 0.29	642	2.66 ± 0.39
BMC spine (g)	754	56.4 ± 12	676	74.3 ± 16
BMC total hip (g)	749	31.7 ± 5.4	669	46.9 ± 8.2
Bone area whole body (m^2^)	711	19.4 ± 1.5	642	23.1 ± 1.8
Bone area, spine (cm^2^)	754	58.3 ± 5.9	676	70 ± 6.9
Bone area, total hip (cm^2^)	749	35.5 ± 3.3	669	46.2 ± 4.8

CSA‐dia = cross sectional area of the radius diaphysis (50% site); BMC = bone mineral content.

The PrCaK‐rich dietary pattern was consistently positively associated with intakes of lowfat milk, lowfat yogurt, fruit, and vegetables, which had the highest positive factor loadings in each year of the survey. Other foods consistently positively associated with this pattern included whole‐meal bread, fish and fish dishes, coffee, and tea. Whereas sugar and preserves, white bread, animal‐based fats, sweet cereal products, processed meats, alcohol, chocolate and confectionery, and savory snacks were consistently negatively associated with the PrCaK‐rich dietary pattern.

Table 4 presents nutrient densities (per 1000 kcal or % of total energy) according to quintiles of PrCaK‐rich dietary pattern *Z*‐scores at age 36 years. In addition to the pattern being rich in protein, calcium, and potassium–containing foods, higher *Z*‐scores for the PrCaK‐rich were associated with lower total energy intakes, greater densities of fiber, minerals (magnesium, phosphorus, and iron) and vitamins (folate, beta‐carotene, vitamin C, vitamin D), and lower densities of carbohydrates and total sugars.

**Table 4:** Energy Intake and Nutrient Densities by Dietary Pattern Quintiles of the Protein‐Calcium‐Potassium–Rich Dietary Pattern at Age 36 Years

	Quintile 1 (*n* = 483)		Quintile 2 (*n* = 482)		Quintile 3 (*n* = 482)		Quintile 4 (*n* = 482)		Quintile 5 (*n* = 482)		
Nutrient intake	Mean ± SD	Range	Mean ± SD	Range	Mean ± SD	Range	Mean ± SD	Range	Mean ± SD	Range	*p* for trend^a^
Dietary pattern *Z*‐score	−1.37 ± 0.53	−4.04 to −0.79	−0.50 ± 0.16	−0.79 to −0.24	−0.02 ± 0.13	−0.24 to 0.19	0.43 ± 0.15	0.19 to 0.71	1.47 ± 0.90	0.71 to 9.31	<0.0001
Energy (kcal)	2719 ± 583	1155–4839	2174 ± 467	1071–4797	1936 ± 516	773–5939	1770 ± 525	515–3924	1581 ± 530	450–4320	<0.0001
Potassium (mg/1000 kcal)	1279 ± 191	790–1892	1410 ± 195	934–2405	1504 ± 212	923–2258	1622 ± 236	1106–2841	1929 ± 443	1112–5223	<0.0001
Calcium (mg/1000 kcal)	350 ± 77	154–667	374 ± 78	152–740	396 ± 85	162–672	429 ± 98	170–822	535 ± 181	140–2680	<0.0001
Protein (%E)	12 ± 1	7–19	14 ± 2	9–22	14 ± 2	9–21	16 ± 2	10–25	18 ± 4	11–38	<0.0001
Fat (%E)	39 ± 5	14–57	39 ± 5	24–54	39 ± 5	22–54	40 ± 5	21–54	39 ± 6	5–55	0.114
Carbohydrate (%E)^b^	45 ± 6	21–61	45 ± 6	18–68	44 ± 6	24–69	42 ± 6	20–61	42 ± 8	10–68	<0.0001
Total sugars (%E)^c^	20 ± 5	7–36	19 ± 6	5–38	18 ± 6	4–49	17 ± 6	3–35	19 ± 6	1–60	<0.0001
NSP fiber (g/1000kcal)	5 ± 1	2–11	5 ± 1	2–12	6 ± 2	2–13	6 ± 2	2–13	8 ± 3	2–28	<0.0001
Magnesium (g/1000 kcal)	125 ± 25	79–223	133 ± 25	84–244	140 ± 27	88–252	150 ± 27	93–253	182 ± 49	104–427	<0.0001
Phosphorus (g/1000 kcal)^d^	513 ± 65	315–771	556 ± 67	384–857	592 ± 71	401–895	641 ± 78	455–971	785 ± 173	455–2281	<0.0001
Iron (mg/1000 kcal)	5 ± 1	3–13	5 ± 1	3–13	6 ± 1	3–18	6 ± 2	4–24	7 ± 2	3–21	<0.0001
Sodium (mg/1000 kcal)^e^	1262 ± 238	630–2001	1279 ± 234	472–1991	1279 ± 251	440–2065	1321 ± 281	665–2361	1410 ± 384	547–5244	<0.0001
Vitamin C (mg/1000 kcal)	24 ± 10	6–91	28 ± 11	7–84	32 ± 13	6–109	37 ± 17	3–190	52 ± 34	8–406	<0.0001
Retinol (g/1000 kcal)^f^	494 ± 680	23–3667	469 ± 788	44–9406	446 ± 720	38–6047	571 ± 1026	43–10878	555 ± 905	0–6262	0.062
Carotene equivalents (g/1000 kcal)^g^	683 ± 485	89–3829	787 ± 542	73–3970	908 ± 598	80–3525	1089 ± 782	57–6264	1381 ± 1159	171–12417	<0.0001
Folate (μg/1000 kcal)	97 ± 35	47–498	99 ± 28	50–263	104 ± 28	50–309	112 ± 29	52–263	133 ± 44	60–439	<0.0001
B12 (μg/1000 kcal)	3 ± 3	0–21	3 ± 4	1–45	3 ± 3	0–27	4 ± 5	1–51	4 ± 5	1–30	<0.0001
Vitamin D (μg/1000 kcal)	1 ± 1	0–5	1 ± 1	0–6	1 ± 1	0–13	1 ± 1	0–8	2 ± 2	0–21	<0.0001

NSP = non‐starch polysaccharides; NMES = non‐milk extrinsic sugars.

^a^ *p* value for trend estimated by modeling dietary pattern quintile as categorical independent variable against nutrient intake.

^b^ Carbohydrates are total carbohydrates (sugars and starch, excluding fiber).

^c^ Total sugars includes both NMES and intrinsic milk sugars (glucose, sucrose, maltose, lactose, fructose, and other sugars).

^d^ Phosphorous intake includes that from processed foods.

^e^ Includes sodium from foods only (not discretionary salt).

^f^ Retinol (g): preformed vitamin A only

^g^ Carotene equivalents (g): in the UK prior to 2012 this included beta carotene equivalents only.^52^

The tracking coefficient for the PrCaK‐rich dietary pattern was 0.34 (95% CI, 0.28 to 0.41) for men and 0.34 (95% CI, 0.29 to 0.39) for women, indicating that this dietary pattern tracked weakly (<0.40) and that an individual's dietary pattern *Z*‐scores were subject to change.

**Figure 2:**
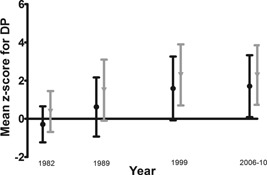
Mean and SD of dietary pattern *Z*‐scores at each time point for males and females. Female (light gray); male (black). DP = dietary pattern.

Individual dietary pattern trajectories (or slopes in dietary pattern *Z*‐scores between age 36 and 60 to 64 years) showed a normal distribution and ranged from –0.002 to 0.23 SD per year, with an average trajectory of 0.07 SD per year (SD = 0.02) (Fig. 2). This indicates that for most participants *Z*‐scores for the PrCaK‐rich dietary pattern increased over the study period (mean dietary pattern *Z*‐score increased by approximately 2 SD; see Fig. 2), and diet quality improved over the period of study.

Multivariate linear regression models showed that a greater positive slope in PrCaK‐rich dietary pattern trajectory, indicating a steeper increase in dietary pattern *Z*‐scores over time, was positively associated with several bone outcomes in women only (Table 5). Because the scale of trajectories was small, we used a base unit of the magnitude equivalent to the standard deviation in dietary pattern trajectories (0.02 SD units) for their interpretation. For example, a 0.02 SD unit/year higher dietary pattern trajectory was associated with a 0.69% (95% CI, 0.07 to 1.31) higher whole‐body SA‐BMC; a 1.40% (95% CI, 0.30 to 2.51) higher spine SA‐BMC; a 1.35% (95% CI, 0.48 to 2.23) higher total hip SA‐BMC; a 1.81% (95% CI, 0.13 to 3.50) higher total vBMD; and 2.13% (95% CI, 0.03 to 4.23) higher trabecular vBMD in women, after adjustment for baseline *Z*‐score and all other available confounders (Model 3, Table 5). A 0.02 SD unit/year higher dietary pattern trajectory between age 36 and 60 to 64 years was negatively associated with medullary area (indicating positive effects on cortical thickness) in women only, but this was not statistically significant after adjustment for lifestyle factors (Table 5). No associations were observed between dietary pattern trajectories and any skeletal outcome in men. There were no statistically significant interactions between dietary pattern *Z*‐score and gender for any of the bone outcomes (*p* > 0.05). Baseline dietary pattern *Z*‐score was not a statistically significant predictor of bone outcome in any of the tested models.

**Table 5:** Associations Between Protein‐Calcium‐Potassium–Rich Dietary Pattern Trajectory (Between Ages 36 to 60‐64 Years) and Skeletal Phenotype at Age 60–64 Years

	Males				Females			
	*n*	beta %^a^	P=	95% CI (%)	*n*	beta %	P=	95% CI (%)
Whole‐body BMC^b^								
Model 1	602	1.60	0.002	0.59 to 2.60	661	0.62	0.238	−0.41 to 1.65
Model 2	602	0.29	0.241	−0.20 to 0.79	661	0.48	0.096	−0.09 to 1.04
Model 3	548	0.18	0.481	−0.32 to 0.69	569	0.69	0.030	0.07 to 1.31
Spine BMC								
Model 1	635	0.92	0.208	−0.51 to 2.35	701	0.77	0.292	−0.66 to 2.20
Model 2	635	0.71	0.142	−0.24 to 1.65	701	1.35	0.009	0.34 to 2.37
Model 3	574	0.47	0.344	−0.50 to 1.45	600	1.40	0.013	0.30 to 2.51
Total hip BMC								
Model 1	628	1.56	0.008	0.40 to 2.72	697	0.45	0.446	−0.71 to 1.61
Model 2	628	0.51	0.197	−0.26 to 1.29	697	1.28	0.002	0.48 to 2.08
Model 3	567	0.09	0.823	−0.71 to 0.89	597	1.35	0.003	0.48 to 2.23
Total density^c^								
Model 1	523	0.37	0.563	−0.88 to 1.62	556	1.62	0.044	0.04 to 3.19
Model 2	523	0.45	0.487	−0.81 to 1.70	556	1.69	0.033	0.14 to 3.24
Model 3	467	0.24	0.718	−1.07 to 1.55	476	1.81	0.035	0.13 to 3.50
CSA radius								
Model 1	525	0.73	0.364	−0.84 to 2.29	565	−1.00	0.064	−2.06 to 0.06
Model 2	525	0.00	0.998	−1.51 to 1.50	565	−0.67	0.174	−1.63 to 0.29
Model 3	470	0.16	0.851	−1.49 to 1.81	484	−0.37	0.487	−1.40 to 0.67
Trabecular density								
Model 1	525	1.41	0.079	−0.16 to 2.98	556	1.73	0.080	−0.21 to 3.67
Model 2	523	1.56	0.051	−0.01 to 3.13	556	1.77	0.070	−0.15 to 3.69
Model 3	467	1.24	0.146	−0.43 to 2.91	476	2.13	0.046	0.03 to 4.23
Medullary area								
Model 1	524	0.82	0.503	−1.59 to 3.24	565	−3.20	0.028	−6.06 to −0.35
Model 2	524	–0.07	0.951	−2.44 to 2.29	565	−2.87	0.045	−5.68 to −0.06
Model 3	469	–0.10	0.937	−2.62 to 2.42	484	−2.50	0.111	−5.58 to 0.57
Polar stress‐strain index								
Model 1	525	1.62	0.032	0.14 to 3.11	565	−0.33	0.658	−1.80 to 1.14
Model 2	525	0.74	0.289	−0.63 to 2.10	565	0.14	0.840	−1.20 to 1.48
Model 3	470	0.67	0.357	−0.76 to 2.10	484	0.62	0.408	−0.85 to 2.09

Data shows the percent difference in outcome per 0.02‐SD change/year in dietary pattern in study participants who completed at least two food diaries during follow‐up, between 1982 and 2009. Model 1: linear regression, with dietary pattern trajectory and the baseline dietary pattern score as the independent variables, all outcomes log‐transformed. Model 2: same as model 1, additionally adjusted for height and weight plus bone area where BMC was the outcome to calculate size‐adjusted BMC (ie, BMD) (all transformed to natural logarithms). Model 3: same as model 2, additionally adjusted for social class, geographic region, physical activity, cigarette smoking, supplement use (calcium, vitamin D, other minerals or multivitamins/minerals), and time since menopause (females).

^a^ Percent difference in outcome associated with a 0.02‐SD unit difference in dietary pattern trajectory.

^b^ Bone mineral content.

^c^ Volumetric bone mineral density.

## Discussion

Using longitudinal dietary data from the oldest‐British post‐war birth cohort study, these data show that, in women, improving the nutrient quality of their diet by increasing their scores for a PrCaK‐rich dietary pattern over adult life (without increasing caloric intake) was positively associated with higher SA‐BMC and vBMD at age 60 to 64 years. Most notably, the associations were strongest at those sites most prone to osteoporotic fracture, the spine and the hip, and were robust to adjustment for a range of important confounders.

The dietary pattern was positively associated with greater intakes of lowfat milk, lowfat yogurt, fruits and vegetables, whole‐meal bread, fish, and fish dishes, and lower intakes of sugar and preserves, white bread, animal‐based fats, sweet cereal products, processed meats, alcohol, chocolate and confectionery, and savory snacks. Importantly, this PrCaK‐rich dietary pattern was also associated with lower total energy intakes, greater densities of fiber, vitamins (folate, carotene, vitamin C, vitamin D), other minerals (magnesium, phosphorus, and iron), and lower densities of carbohydrate and total sugars. The pattern identified broadly agreed with previous observational studies in which “nutrient dense” patterns were positively associated with BMC, BMD, bone turnover markers, or fracture risk.^6^, ^7^, ^9^, ^10^, ^14^, ^19^ In the majority of studies, “nutrient dense” denotes a pattern rich in fruits and vegetables, and whole grains, with low consumption of processed and sugary foods. The limitations of those studies were that the methodology used did not allow specific nutrient or food groups to be identified as contributing to bone health, and that they were conducted at one time point so the importance of changing diet could not be related to bone outcomes. As recently noted by Hannan and colleagues^2^ in a commentary in the *Journal of Bone and Mineral Research*, studies such as the one presented here are needed to fill a gap in understanding of the relationship between diet and health outcomes by extending the analysis beyond single micronutrients or macronutrients to describe whole diet with respect to bone health.

The findings from this study are in agreement with previous studies that showed positive associations between dietary patterns characterized by greater consumption of fruit, vegetables, and whole grains and BMD in females.^6^, ^9^, ^10^, ^13^, ^14^, ^15^, ^16^, ^17^, ^18^ In contrast, combinations of unhealthy foods, including fried food, savory pies, confectionery, soft drinks, red and processed meats, and biscuits (cookies), have been negatively associated with BMD/BMC.^6^, ^7^, ^13^, ^18^ There are few studies that have prospective fracture data. Data from the Canadian Multicentre Osteoporosis Study (CAMOS) cohort showed that a healthy pattern was associated with lower risk of low‐trauma fractures in postmenopausal women (hazard ratio 0.86; 95% CI, 0.76 to 0.9).^5^ In two Swedish cohorts higher rates of hip fracture in women were associated with a fruit and vegetable intake of less than five portions per day; less than one serving per day was associated with a 50% increased risk of fracture (hazard ratio 1.49; 95% CI, 1.32 to 1.68).^19^ The PrCaK‐rich dietary pattern identified in the current study supports current guidelines produced for the prevention of osteoporosis.^49^ More broadly, this pattern is similar to the dietary pattern described in the American Heart Association Diet and Lifestyle recommendations for reducing cardiovascular disease risk, which was also associated with reduced risk of fracture and increased BMD.^14^ Translating any advice from studies to public health guidelines is much easier for the public to interpret if there is a consistent message across common health conditions.^20^, ^50^

At the population level, the tracking of scores for the PrCaK‐rich dietary pattern was not strong. This is not surprising, because the results from the dietary pattern trajectories showed that *Z*‐scores were not constant, but tended to increase over time. These increases between age 36 years and 60 to 64 years indicate improvements in diet quality that were concurrent with changes in the UK food supply (eg, increased availability of lowfat dairy, and other products)^35^ and the emergence of public health messages regarding diets for the prevention of chronic disease; eg, reduce fat intakes, avoid solid animal fats, consume more fruits and vegetables.

In contrast to our observations in women, no associations were observed between the dietary pattern and bone outcomes in men. This may be because the men were at a different stage of skeletal aging than the women. This is supported by studies of bone during male aging that suggest skeletal aging in males occurs more slowly, and starts at around age 60 years when hormonal changes start to occur.^51^ However, in the Swedish cohort study positive associations were reported between dietary patterns and reduced fracture risk in men,^19^ and in the CAMOS study there was a positive trend toward significance.^5^ Given these previous findings, and those in our cohort in women, it will be important to investigate whether these relationships emerge in men at an older age when age‐related bone loss has progressed farther and to investigate alternative dietary patterns. Another contributing factor may be that men in our study showed smaller changes in mean *Z*‐scores for the PrCaK‐rich dietary pattern over time than women (Fig. 2).

By including pQCT as an outcome measure we were also able to explore whether there were associations between dietary patterns and other aspects of bone health at the peripheral skeleton; ie, bone size, distribution, and strength. A greater improvement in the dietary score was associated with smaller medullary area but not with total area or strength. These data indicate that an increase in the consumption of protein, calcium, and potassium–rich foods during adulthood was associated with less endosteal resorption and thus reduced cortical thinning, which would be protective against bone fragility. These data support evidence from previous studies that have shown reduced hip fracture risk in those with better diets.^5^, ^19^

There are several strengths to this study. The availability of longitudinal dietary assessments from multiple time points collected over 28 years using the same methodology and contemporaneous food composition data provided a opportunity to examine dietary patterns over the life course. The use of longitudinal models exploited all available data rather than limiting the analysis to respondents who completed all follow‐ups, thus minimizing the possibility of a selective sample. Detailed dietary data were collected in the form of un‐weighed 5‐day or 7‐day food diaries that provided information on the both the range and combinations of foods in this cohort, enabling detailed dietary pattern analyses. Our chosen method of dietary pattern analysis, RRR, allowed us to develop a hypothesis based on previous studies in which micronutrients and macronutrients were related to bone health and to identify patterns in food consumption that best explained provision of these nutrients in this cohort. Our bone outcomes were available from DXA and pQCT measurements at multiple skeletal sites. Finally, the sample size was moderately large across time points, and because of the nature of the cohort data collection and the narrow age‐range at each time point, which minimized confounding by age, we were able to adjust for multiple confounders and lifestyle factors across adulthood. The main limitation of this study was the age of the population who are at a relatively early stage of aging; also, at age 60 to 64 years rates of osteoporosis and osteopenia in the population were low. Continuing follow‐up in this cohort into old‐age will be important to ascertain whether this dietary pattern predicts reductions in age‐related bone loss in individuals and fracture incidence by making both the exposure and outcome data longitudinal.

In conclusion, a nutrient‐dense dietary pattern that is rich in protein, calcium, and potassium associated with lower energy intake during adulthood is associated with better bone health at fracture prone sites in women. Such a dietary pattern is characterized by greater intakes of lowfat milk and yogurts, whole‐grain bread and breakfast cereals, fruits and vegetables, and lower intakes of sugars, sweets, processed foods, and animal fats. To increase scores for this PrCaK‐rich dietary pattern while maintaining total energy intake and to achieve a net improvement in overall diet quality, intakes of foods positively associated with the pattern would need to increase and intakes of foods negatively associated with the pattern would need to decrease. The findings of this dietary pattern analysis support current public health dietary guidelines for the dietary prevention of osteoporosis.

## Disclosures

All authors state that they have no conflicts of interest.

## Supporting information

Supporting Information.
