# Supplementary material for: Life Course Dietary Patterns and Bone Health in Later Life in a British Birth Cohort Study
Source: J Bone Miner Res. 2016 Mar 8;31(6):1167–76. doi: 10.1002/jbmr.2798 (PMC4982044; doi:10.1002/jbmr.2798)
Supplement: Supplementary file 1 — Supporting Information. [file JBMR-31-1167-s001.docx]

## **Supporting Table 1: A description of food groups used in DINO**

| Low fat Milk | Skimmed and semi-skimmed milk |
| --- | --- |
| Whole Milk | Whole milk, Milk based drinks, e.g. flavoured milks |
| Cheese & Cream | Cheese incl. cottage cheese, cream & fromage frais |
| Ice cream | Ice cream & dairy desserts, full and reduced fat |
| Full Fat Yoghurt | full fat yoghurt & drinking yoghurts, incl. buttermilk |
| Low fat Yoghurt | Reduced and low fat dairy products - Yoghurt & drinking yoghurts, incl. buttermilk |
| Fruit | Fresh, dried, canned and cooked fruit |
| Vegetables | Raw, canned, cooked incl. tomatoes and leafy green veg |
| Potatoes | Potatoes and potato products incl. chips, gnocchi, potato waffles |
| White bread | White breads and rolls |
| Wholemeal bread | Brown, granary, wheatgerm and wholemeal breads, wholemeal crispbreads |
| High fibre breakfast cereals | Oat based cereals, other breakfast cereals (fibre equal or >3g/40g portion) |
| Low fibre breakfast cereals | Other breakfast cereals (not categorised as high fibre) |
| Other cereal and cereal dishes | Other cereals (not incl. pizza, pasta, rice) & cereal dishes e.g. yorkshire puddings, instant noodle pots, stuffing, savory scones, dumplings, batter, pastry and plain pancakes |
| Cereal bars | Sweet cereal products incl. cereal and muesli bars |
| Pasta & pasta dishes | Pasta & pasta dishes |
| Rice & rice dishes | Rice & rice dishes |
| Pizza | Pizza |
| Sugar and preserves | Pure added sugar, jams and preserves |
| Syrups, honey | Sugar syrups incl. honey |
| Chocolate confectionary | Chocolate based products |
| Sugar confectionary | Sorbets & lollies, sugar based confectionery |
| Sweet cereal products | Sweet pastries, buns and pies, biscuits, cereal based puddings (not milk), milk based puddings |
| Savoury Snacks | Cereal based snacks, Potato based snacks, Savoury biscuits & crackers, Vegetable based snacks incl. crisps |
| Egg & egg dishes | Egg & egg dishes |
| Pulses Lentils | Baked beans, Pulses, Lentils |
| Red meat | Beef, veal, lamb, game and other red meat dishes, Pork dishes |
| Processed Meat | Bacon, ham and other processed meats, processed meat pies, sausages, burgers & kebabs |
| White meat | Chicken, turkey, game bird (e.g. duck, goose, pheasant) dishes |
| Fish & fish dishes | Oily fish, Shellfish, White fish dishes, incl. tuna |
| Offal | Liver & Other offal e.g. Haggis, faggots |
| Soups | Canned, fresh, homemade and dried (instant) soups |
| Animal based fats | Butter, lard and other animal (hard ) fats |
|  |  |
| Plant based fats | Plant based margarines, incl. low fat and reduced fat |
| Oils | Vegetable and cereal-based oils ( |
| Nuts & Seeds | Nuts & Seeds (incl. peanut butter) |
| Chutney & Pickles | Chutney, Pickles (incl. gherkins, pickled onions etc) |
| Sauces & accompaniment | Cooking sauces, incl. gravies, pesto, cooking sauces for pasta and rice dishes, Dressings & Mayonnaise, Other sauces, incl. brown sauce, soy sauce, ketchup, mint sauce, vinegar |
| Alcohol | Alcopops, Beer (incl. low alc), Fortified wine, Low alcohol beer, Spirits & Liqueur |
| Wine | Red and white wines |
| Soft Drinks | Carbonated soft drinks |
| Coffee & Tea | Tea, Coffee, Powdered Beverages (cocoa, Horlicks, Bonvita, Ovaltine, etc) |
| Fruit based drinks | Fruit based drinks, pure fruit juice & smoothies |
| Squashes and fruit concentrates | Squashes & fruit concentrates |
| Nutrition Powders & drinks | Nutrition Powders & drinks |
| Miscellaneous | Artificial sweeteners, Dried herbs & spices & pastes  Salt and salt substitutes |

**Supporting Fig. 1**. Factor Loadings for the protein, calcium and potassium‑rich dietary pattern in 1989, 1999, 2006-10
